# Supplementary material for: The Gut Bacteria Dysbiosis Contributes to Chronic Graft-Versus-Host Disease Associated With a Treg/Th1 Ratio Imbalance
Source: Front Microbiol. 2022 Sep 8;13:813576. doi: 10.3389/fmicb.2022.813576 (PMC9493085; doi:10.3389/fmicb.2022.813576)
Supplement: Supplementary file 1 [file Data_Sheet_1.zip › P101SC18090073-01-B1-3-4_result/05.WebShow/taxa_charts_group_html/charts/GWQ00KpEPgzCPRWzFY4H2qZ0Qu4UFL_legend.pdf]

- k__Archaea;p__Euryarchaeota;c__Methanobacteria
- k__Bacteria;Other;Other
- k__Bacteria;p__Acidobacteria;c__Holophagae
- k__Bacteria;p__Acidobacteria;c__unidentified_Acidobacteria
- k__Bacteria;p__Actinobacteria;c__Acidimicrobiia
- k__Bacteria;p__Actinobacteria;c__Coriobacteriia
- k__Bacteria;p__Actinobacteria;c__Rubrobacteria
- k__Bacteria;p__Actinobacteria;c__Thermoleophilia
- k__Bacteria;p__Actinobacteria;c__unidentified_Actinobacteria
- k__Bacteria;p__Armatimonadetes;c__Fimbriimonadia
- k__Bacteria;p__Bacteroidetes;c__Bacteroidia
- k__Bacteria;p__Deinococcus-Thermus;c__Deinococci
- k__Bacteria;p__Firmicutes;Other
- k__Bacteria;p__Firmicutes;c__Bacilli
- k__Bacteria;p__Firmicutes;c__Clostridia
- k__Bacteria;p__Firmicutes;c__Erysipelotrichia
- k__Bacteria;p__Firmicutes;c__Negativicutes
- k__Bacteria;p__Fusobacteria;c__Fusobacteriia
- k__Bacteria;p__Melainabacteria;Other
- k__Bacteria;p__Oxyphotobacteria;c__unidentified_Oxyphotobacteria
- k__Bacteria;p__Proteobacteria;Other
- k__Bacteria;p__Proteobacteria;c__Alphaproteobacteria
- k__Bacteria;p__Proteobacteria;c__Deltaproteobacteria
- k__Bacteria;p__Proteobacteria;c__Gammaproteobacteria
- k__Bacteria;p__Tenericutes;c__Mollicutes
- k__Bacteria;p__Verrucomicrobia;c__Verrucomicrobiae
- k__Bacteria;p__unidentified_Bacteria;c__unidentified_Bacteria
